# Supplementary material for: A Critical View of the Application of the APEX Software (Aqueous Photochemistry of Environmentally-Occurring Xenobiotics) to Predict Photoreaction Kinetics in Surface Freshwaters
Source: Molecules. 2019 Dec 18;25(1):9. doi: 10.3390/molecules25010009 (PMC7017383; doi:10.3390/molecules25010009)
Supplement: Supplementary file 1 [file molecules-25-00009-s001.zip › 3.2.4_gcc-4.4.0.zip!/3.2.4_gcc-4.4.0/3.2.4_gcc-4.4.0/doc/octave/html/interpreter/Products-of-Polynomials.html]

Products of Polynomials - Untitled


Next: Derivatives and Integrals,
Previous: Finding Roots,
Up: Polynomial Manipulations


---

### 27.3 Products of Polynomials

— Function File: **conv** (a, b)  
> Convolve two vectors.
>
> `y = conv (a, b)` returns a vector of length equal to
> `length (a) + length (b) - 1`.
> If a and b are polynomial coefficient vectors, `conv`
> returns the coefficients of the product polynomial.
>
>
>
> **See also:** deconv, poly, roots, residue, polyval, polyderiv, polyinteg.

— Function File: c = **convn** (a, b, shape)  
> N-dimensional convolution of matrices a and b.
>
> The size of the output is determined by the shape argument.
> This can be any of the following character strings:
>
> "full": The full convolution result is returned. The size out of the output is `size (`a`) + size (`b`)-1`. This is the default behavior. "same": The central part of the convolution result is returned. The size out of the output is the same as a. "valid": The valid part of the convolution is returned. The size of the result is `max (size (`a`) - size (`b`)+1, 0)`.
>
>
>
>
> **See also:** conv, conv2.

— Function File: **deconv** (y, a)  
> Deconvolve two vectors.
>
> `[b, r] = deconv (y, a)` solves for b and r such that
> `y = conv (a, b) + r`.
>
> If y and a are polynomial coefficient vectors, b will
> contain the coefficients of the polynomial quotient and r will be
> a remainder polynomial of lowest order.
>
>
>
> **See also:** conv, poly, roots, residue, polyval, polyderiv, polyinteg.

— Loadable Function: y = **conv2** (a, b, shape)  
— Loadable Function: y = **conv2** (v1, v2, M, shape)  
> Returns 2D convolution of a and b where the size
> of c is given by
>
> shape= 'full': returns full 2-D convolution shape= 'same': same size as a. 'central' part of convolution shape= 'valid': only parts which do not include zero-padded edges
>
> By default shape is 'full'. When the third argument is a matrix
> returns the convolution of the matrix M by the vector v1
> in the column direction and by vector v2 in the row direction

— Function File: q = **polygcd** (b, a, tol)  
> Find greatest common divisor of two polynomials. This is equivalent
> to the polynomial found by multiplying together all the common roots.
> Together with deconv, you can reduce a ratio of two polynomials.
> Tolerance defaults to
>
> ```
>           sqrt(eps).
> ```
>
> Note that this is an unstable
> algorithm, so don't try it on large polynomials.
>
> Example
>
> ```
>           polygcd (poly(1:8), poly(3:12)) - poly(3:8)
>           => [ 0, 0, 0, 0, 0, 0, 0 ]
>           deconv (poly(1:8), polygcd (poly(1:8), poly(3:12))) ...
>             - poly(1:2)
>           => [ 0, 0, 0 ]
> ```
>
>
>
>
> **See also:** poly, polyinteg, polyderiv, polyreduce, roots, conv, deconv, residue, filter, polyval, polyvalm.

— Function File: [r, p, k, e] = **residue** (b, a)  
> Compute the partial fraction expansion for the quotient of the
> polynomials, b and a.
>
> ```
>            B(s)    M       r(m)         N
>            ---- = SUM -------------  + SUM k(i)*s^(N-i)
>            A(s)   m=1 (s-p(m))^e(m)    i=1
> ```
>
> where M is the number of poles (the length of the r,
> p, and e), the k vector is a polynomial of order N-1
> representing the direct contribution, and the e vector specifies
> the multiplicity of the m-th residue's pole.
>
> For example,
>
> ```
>           b = [1, 1, 1];
>           a = [1, -5, 8, -4];
>           [r, p, k, e] = residue (b, a);
>                => r = [-2; 7; 3]
>                => p = [2; 2; 1]
>                => k = [](0x0)
>                => e = [1; 2; 1]
> ```
>
> which represents the following partial fraction expansion
>
> ```
>                   s^2 + s + 1       -2        7        3
>              ------------------- = ----- + ------- + -----
>              s^3 - 5s^2 + 8s - 4   (s-2)   (s-2)^2   (s-1)
> ```
>
> — Function File: [b, a] = **residue** (r, p, k)  
> — Function File: [b, a] = **residue** (r, p, k, e)  
> > Compute the reconstituted quotient of polynomials,
> > b(s)/a(s), from the partial fraction expansion;
> > represented by the residues, poles, and a direct polynomial specified
> > by r, p and k, and the pole multiplicity e.
> >
> > If the multiplicity, e, is not explicitly specified the multiplicity is
> > determined by the script mpoles.m.
> >
> > For example,
> >
> > ```
> >           r = [-2; 7; 3];
> >           p = [2; 2; 1];
> >           k = [1, 0];
> >           [b, a] = residue (r, p, k);
> >                => b = [1, -5, 9, -3, 1]
> >                => a = [1, -5, 8, -4]
> >           
> >           where mpoles.m is used to determine e = [1; 2; 1]
> > ```
> >
> > Alternatively the multiplicity may be defined explicitly, for example,
> >
> > ```
> >           r = [7; 3; -2];
> >           p = [2; 1; 2];
> >           k = [1, 0];
> >           e = [2; 1; 1];
> >           [b, a] = residue (r, p, k, e);
> >                => b = [1, -5, 9, -3, 1]
> >                => a = [1, -5, 8, -4]
> > ```
> >
> > which represents the following partial fraction expansion
> >
> > ```
> >               -2        7        3         s^4 - 5s^3 + 9s^2 - 3s + 1
> >              ----- + ------- + ----- + s = --------------------------
> >              (s-2)   (s-2)^2   (s-1)          s^3 - 5s^2 + 8s - 4
> > ```
> >
> >
> >
> >
> > **See also:** poly, roots, conv, deconv, mpoles, polyval, polyderiv, polyinteg.
